# Low Temperature Hot Corrosion Screening of Single Crystal Superalloys

**DOI:** 10.3390/ma11112098

**Published:** 2018-10-25

**Authors:** James L. Smialek, Simon Gray

**Affiliations:** 1 NASA Glenn Research Center, Cleveland, OH 44135, USA; 2 Surface Engineering & Nanotechnology Institute, Cranfield University, Bedfordshire MK43 0AL, UK; s.gray@cranfield.ac.uk

**Keywords:** Type II hot corrosion, single crystal superalloys, molten salt

## Abstract

Single crystal superalloys were screened in Type II molten (Na,K)-sulfate hot corrosion re-coat tests in air +300 ppm SO_2_ at 700 °C. They exhibited large 20–40 mg/cm^2^ weight changes, repeated spallation, and non-protective, 25–50 μm thick corrosion layers after 300 h of testing. Scale cross sections revealed dual outer Ni(Co)O and inner Al(Cr)S-rich corrosion layers. This chemical differentiation was partially consistent with previous models of oxide fluxing, alloy sulfidation, NiO micro-channel diffusion, and synergistic dissolution mechanisms. Broad shallow pits or uniform attack morphologies were consistent with prior studies performed in high >100 ppm pSO_2_ environments. Higher Mo experimental alloys trended toward more degradation, producing 100 μm thick scales with distinct Al(Cr)S-rich inner layers or 500 μm thick NiO. The aggressive behavior in these environments supports the need for LTHC-resistant coatings for single crystal superalloys.

## 1. Introduction

Low temperature hot corrosion (LTHC) is a recurring concern for Ni-base superalloy components, especially now for advanced disk applications envisioned up to 815 °C (1500 °F). Here it can be expected that Ni(Co)SO_4_-Na_2_SO_4_ eutectic salts may be formed as low as 660 °C (585 °C) with the potential for aggressive corrosion as well as strength-limiting corrosion pits, where low cycle fatigue debits up to 92% have been reported due to 760 °C LTHC [1]. Accordingly, serious effort has been put forth to investigate corrosion resistant Ni-Cr-Y sputter coatings and their ability to diminish the corrosion pitting deficit [1]. The use of a corrosion resistant Cr_2_AlC MAX phase compound has also been explored for this application [2,3,4]. Damage tolerance and thermal expansion matching with superalloys were viewed as other positive attributes of this MAX phase.

In a recent LTHC screening test, Cr_2_AlC MAX phase samples and the NASA LSHR (low solvus high refractory) disk alloy were exposed to repeated salt coatings and exposed to 700 °C air with 300 ppm SO_2_, using 25 h cycles [4]. Weight change and appearance were followed with time up to 500 h. Elemental rasters were obtained for polished cross sections of samples exposed for 300 h. In general, the Cr_2_AlC samples produced moderate weight gains with some evidence of accelerated oxidation. The LSHR superalloy exhibited larger gains, then losses as thick corrosion layers repeatedly spalled and regrew. The elemental rasters also revealed distinct sulfur concentrations mixed within the scales, indicating some accelerating role of the salt for both systems. Furthermore, the LSHR samples revealed a banded outer Ni(Co) oxide and inner Cr(S) scale morphology, more typical of oxide fluxing–re-precipitation LTHC mechanisms.

LTHC can also be a problem for lower temperature portions of turbine blades, typically Ni-base single crystal superalloys (SXSA). These regions consist of the blade root structures, such as the fir tree attachments to the disks and blade platforms. Another design aspect of the advanced turbine disk program entails a hybrid structure, where the outer rim of the disk is constructed of welded segments of single crystal alloys. This is to take advantage of the higher temperature creep strength of cast single crystals as compared to the intermediate temperature fatigue strength of the forged PM disk polycrystalline material. One initial program effort proposed using NASA LDS (Low Density Superalloy) alloys. These are optimized high-Mo (no W) alloys developed by MacKay et al. [5]. More recently, emphasis has been placed on the SC 180 alloy for partnership with Honeywell turbine engines. The main purpose of the present study was to perform the same LTHC screening tests on single crystals as were done for the Cr_2_AlC and LSHR materials and examine the severity of corrosion attack compared to that of the disk alloy. Another purpose was to highlight any differences observed for the high (7–12 wt. %) Mo LDS alloys, since Mo is a known player in alloy-induced acidic hot corrosion mechanisms. Commercial Rene′N5, CMSX-4^®^, and SC 180 single crystal superalloys were also tested, but these have only 0.6–2.0% Mo.

In general, the commercial Gen II single crystals possess among the best cyclic oxidation resistance of all superalloys because of their ability to form an adherent slow growing inner layer of α-Al_2_O_3_. The best LDS alloys were somewhat comparable in 1100 °C, 200 h cyclic oxidation tests, but slightly less protective [6]. Here, the final weight changes for 5% Cr LDS alloys were −1 to +1 mg/cm^2^ compared to −1 and +0.5 mg/cm^2^ for Rene′N4 and Rene′N5, respectively. Scale phases commonly identified were Al_2_O_3_, NiAl_2_O_4_, NiTa_2_O_6_ and NiO, with (Ni,Co)MoO_4_ found only on the least resistant alloys having 0% Cr and 12% Mo. The 5Cr-10Co-7Mo-3Re 1101 LDS alloy, based on a high Mo, low W variation of Rene′N5, possessed the best balance of creep, density, phase stability, and oxidation resistance [7]. Furthermore, Mach 0.3 burner rig hot corrosion also identified nearly equivalent Type I 900 °C hot corrosion resistance for the 1101 LDS alloy compared to Rene′N5, CMSX-4, and CMSX-10 over the 200 h test [7]. Detailed descriptions of various recent LTHC studies will be covered in the Discussion, but are not intended as reviews of the classic literature on the subject (e.g., Luthra, Rapp, Pettit, Meier, Misra, etc.).

## 2. Experimental Section

Single crystal Ni-base superalloy ingots were obtained from commercial processing vendors. The nominal compositions are listed in Table 1 along with that for the LSHR disk alloy. In general, the single crystals contained 5–6% Al, 5–7% Cr, 5–6% W, 6–7% Ta, 2–12% Mo, and 0–3% Re (by wt.). The commercial alloys contained 0.1–0.2% Hf, while the experimental LDS alloys contained 50–100 ppmw Y.

Low temperature, Type II hot corrosion (LTHC) was performed exactly as that in the companion study [4]. Small, roughly 1 × 5 × 10 mm samples were polished to a 2400 grit emery finish and ultrasonically cleaned in ethanol. A saturated aqueous 20 K_2_SO_4_-80 Na_2_SO_4_ salt mixture (mole %, T_eutectic_ ≈ 823 °C) was sprayed on heated samples, weighed, giving one side coated with ~0.5 mg/cm^2^ of salt every 50 h of testing. This ‘flux’ corresponds to high rates (10 μg/cm^2^/h) that produced early propagation in identical tests of CMSX-4 [8]. The LTHC test was conducted at 700 °C in air-300 vppm SO_2_ (flow rate of 50 cm^3^/min) and cycled every 25 h. Duplicate samples were employed and removed from test at 100, 200, 300, up to 500 h, weighed and photographed. After 300 h, one set of corroded samples was mounted in epoxy and polished with non-aqueous media to retain water soluble salts and corrosion products, then examined by optical microscopy and SEM/EDS elemental raster mapping (Philips XL40 SFEG, Eindhoven, The Netherlands). Further experimental test details can be found in prior works [9,10,11].

## 3. Results

### 3.1. Overall Comparison

**Figure 1:**
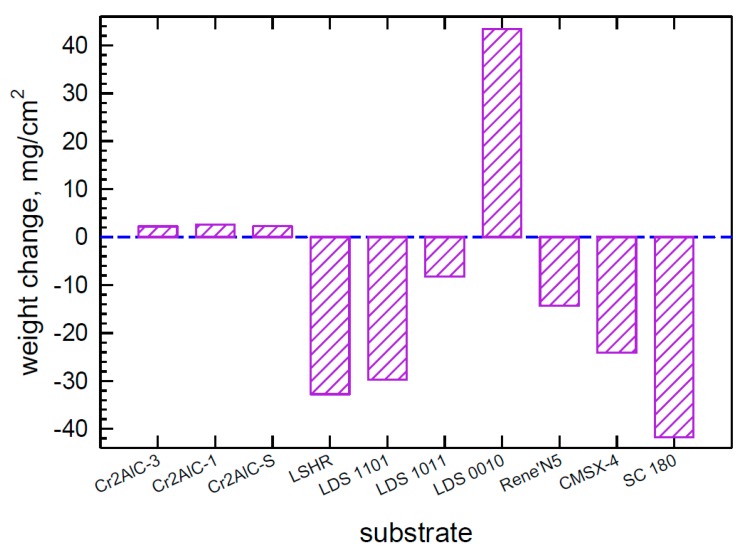
Bar chart comparing final 300 h weight change of Cr_2_AlC, LSHR disk alloy, and the six present single crystal superalloys (SXSA) in LTHC re-coat furnace tests at 700 °C. (80Na-20K)_2_SO_4_ salt spray, 0.5 mg/cm^2^ every 50 h, 25 h heating cycles. pSO_2_ = 300 ppm in air. Includes results from Reference [4].

An overview of the global LTHC response behavior can be seen in Figure 1 for the single crystal superalloys of this work and the Cr_2_AlC samples and LSHR disk superalloy from the previous report [4]. Here the weight change of samples tested to 300 h can be readily compared. First, the Cr_2_AlC samples all show slight weight gains, discussed previously as a combination of scale growth and salt deposits, with minimal spallation. The SXSA single crystal superalloys (LDS, N5, CMSX-4, SC 180) show a wide dispersion of values, most often losses at the end of testing. The absolute value in many cases is as high as 20–40 mg/cm^2^, as compared to only ~3 mg/cm^2^ for Cr_2_AlC. The significance of the wide variations became clearer as the individual weight change curves and macrostructures are examined below.

### 3.2. Weight Change Plots

**Figure 2:**
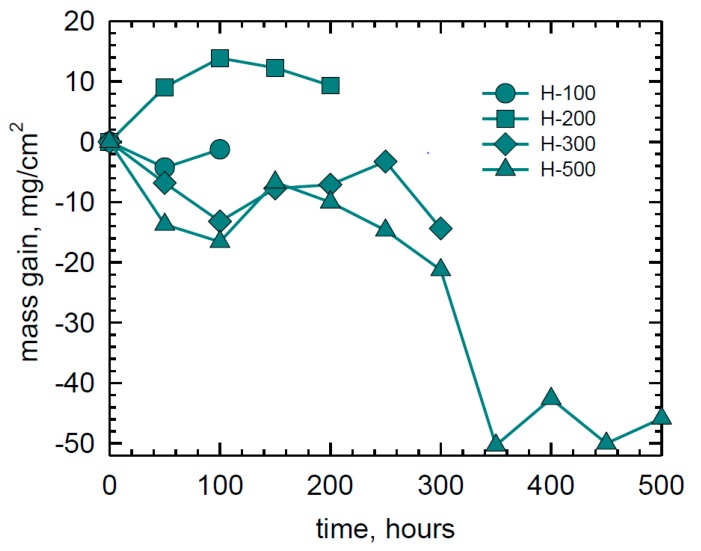
Weight change behavior of duplicate Rene′N5 SXSA samples in LTHC re-coat furnace tests at 700 °C showing large gains and losses. (80Na-20K)_2_SO_4_ sulfate salt spray, 0.5 mg/cm^2^ every 50 h, 25 h cycles. pSO_2_ = 300 ppm in air.

**Figure 3:**
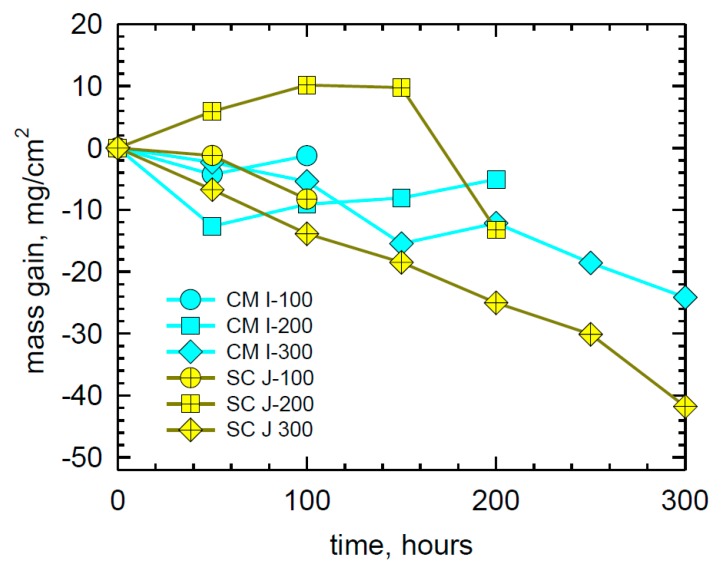
Weight change behavior of duplicate CMSX-4 SXSA samples showing significant losses and SC 180 showing larger gains and losses in LTHC re-coat furnace tests at 700 °C. (80Na-20K)_2_SO_4_ sulfate salt spray, 0.5 mg/cm^2^ every 50 h, 25 h cycles. pSO_2_ = 300 ppm in air.

The weight change behavior of four (H) Rene′N5 samples up to 500 h is shown in Figure 2. Large bifurcations between gains of +15 and losses of −50 mg/cm^2^ were produced. The gain/loss trend changed multiple times and in different directions, depending on the sample. The weight change of three (I) CMSX-4 and (J) SC180 samples are shown in Figure 3. Bifurcations similar to those of N5 are seen, but with less overall magnitude for CMSX-4.

**Figure 4:**
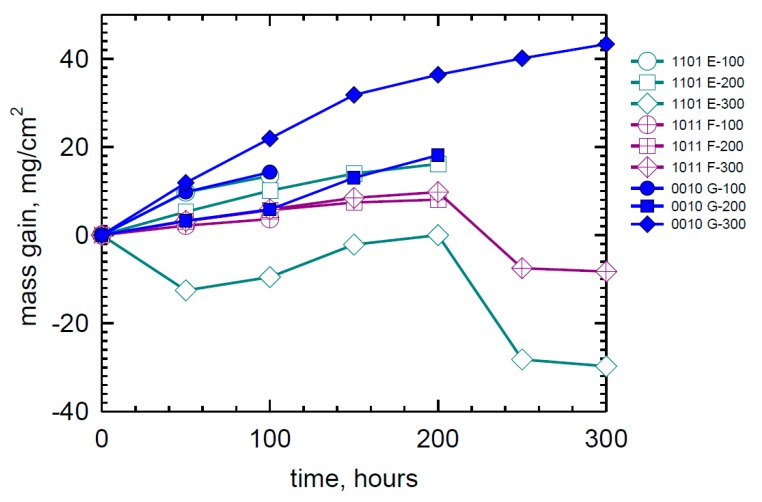
Weight change behavior of duplicate high-Mo LDS SXSA samples in LTHC re-coat furnace tests at 700 °C showing large gains or large losses for specific alloys. See Table 1 for alloy coding. (80Na-20K)_2_SO_4_ sulfate salt spray, 0.5 mg/cm^2^ every 50 h, 25 h cycles. pSO_2_ = 300 ppm in air.

The weight change of the three LDS SXSA is given in Figure 4. The LDS (E)1101 (10Co-5Cr-7Mo-3Re) alloy samples show both substantial weight gain and weight loss depending on the sample. The data spread is a bit tighter for the LDS (F) 1011 alloy (10Co-0Cr-12Mo-3Re), losing a maximum of ~8 mg/cm^2^ after 300 h. Finally, the (G) 0010 samples (0Co-0Cr-12Mo-0Re) exhibit primarily weight gains, achieving the most of any sample at ~43 mg/cm^2^.

### 3.3. Macrographic Appearance

**Figure 5:**
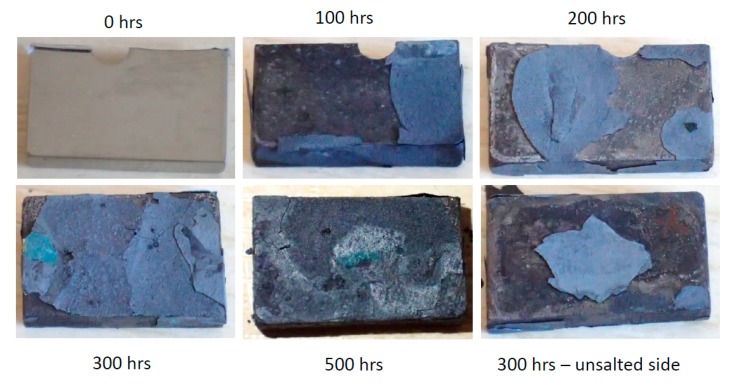
Macrographs showing visual appearance of aggressive attack and spallation for corroded Rene′N5 as a function of time.

**Figure 6:**
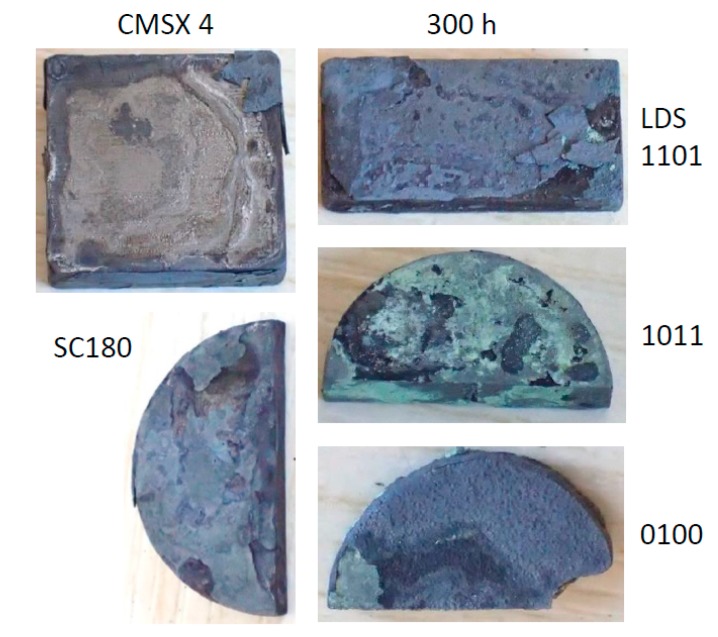
Macrographs showing visual appearance of aggressive attack and spallation for corroded CMSX-4, SC180, and three LDS alloys after 300 h.

The surfaces of Rene′N5 (Figure 5) exhibits large flakes of blue scale, with evidence of massive spallation events occurring throughout the test sequence. This occurred even on the uncoated underside, which indicates salt ‘creep’ or flow around and under the sample. The surfaces of the other alloys after 300 h of LTHC testing are summarized in Figure 6. The (I) CMSX-4 samples show fully spalled blue corrosion scales. Other macrographs (not shown) revealed that the underside was again completely corroded. The surfaces of (J) SC180 showed variations of the prior two alloys, with perhaps less underside salt flow and corrosion at 300 h.

The LDS alloys similarly showed substantial blue corrosion scales, but with more intact surfaces and less partially spalled brittle layers. It was clear from other images that again substantial corrosion occurred from the backsides.

### 3.4. 300 h Cross Sections and Elemental Rasters

#### 3.4.1. Commercial SX Superalloys

**Figure 7:**
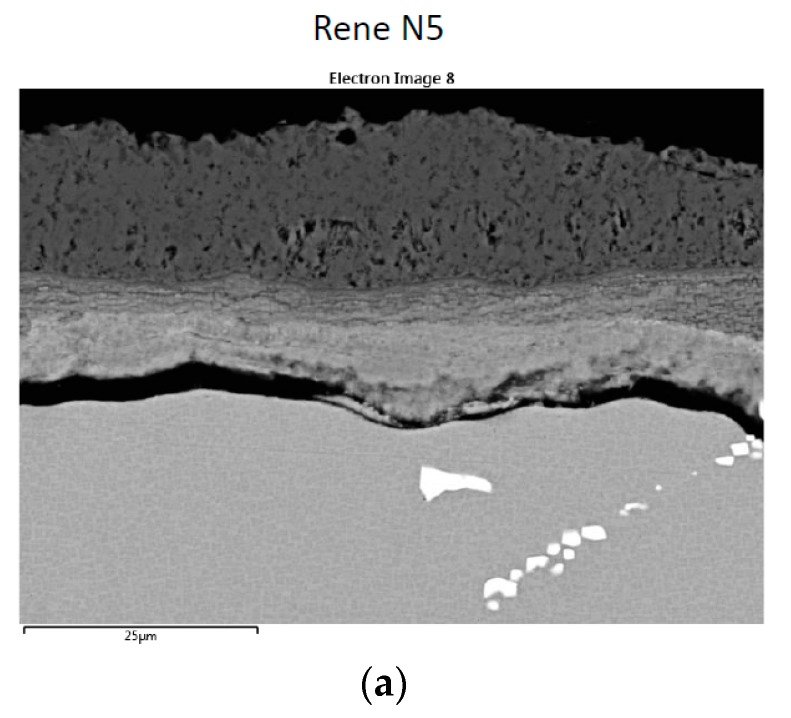

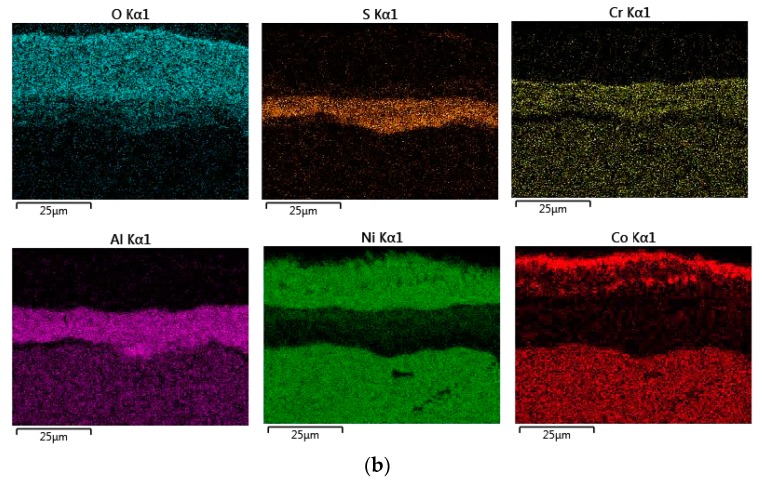
Cross-section of Rene′N5 sample corrosion layers after 300 h LTHC at 700 °C. (**a**) Secondary electron (SE) image showing two primary layers and internal carbides. (**b**) Elemental rasters for O, S, Cr, Al, Ni, Co showing inner Cr, Al, S bands and outer Ni, Co bands.

**Figure 8:**
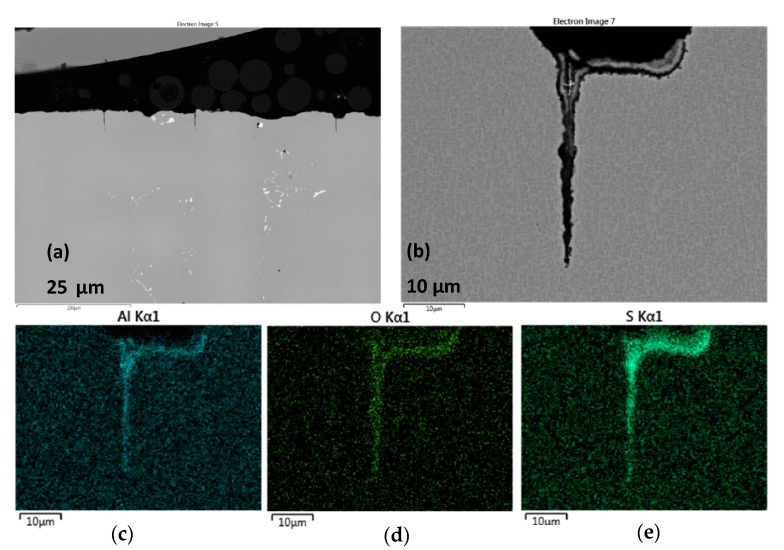
Cross-section of Rene′N5 sample after 300 h LTHC at 700 °C showing deep crack-like penetrations ((**a**,**b**) SE images) with Al, O, S concentration ((**c**–**e**) elemental rasters).

The corrosion scale for alloy (H) Rene′N5 is shown in Figure 7. It exhibits dual layers of approximately 20 + 25 µm, having a wavy attack morphology, but not a pit. Bright Ta(W) borides (carbides) also decorated the alloy microstructure. The inner bands appear to be Al(Cr)-O, with additional Al(Cr)-S concentration at the immediate alloy interface. No Na intensity (residual sulfate) was observed in the scales formed on the superalloys in this study. Low intensity (black) interfacial portions indicate delamination of the scale. The outer layer is primarily Ni-O, with a Co-O external surface, similar to that shown for the LSHR alloy in our previous report [4]. 25 µm deep Al, S, O penetrations down vertical cracks were also observed, as illustrated in Figure 8.

**Figure 9:**
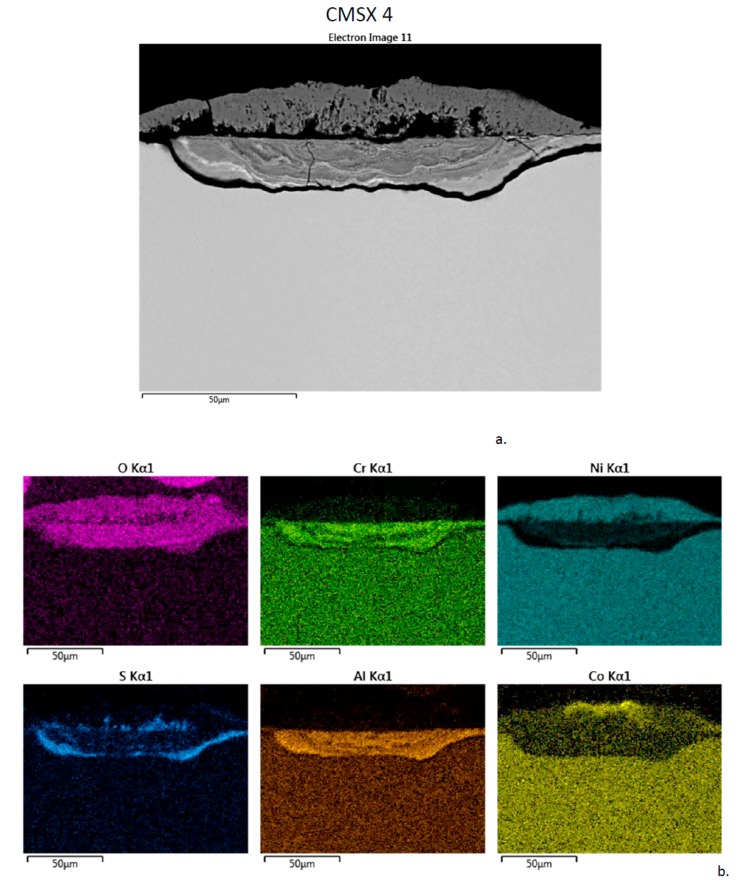
Cross-section of CMSX-4 sample of outer corrosion nodule and inner shallow corrosion pit after 300 h LTHC at 700 °C. (**a**) SE image showing two primary layers. (**b**) Elemental rasters for O, Cr, Ni, S, Al, Co showing inner Cr, Al, S bands in pit and outer Ni, Co bands in nodule.

The corrosion product produced on (I) CMSX-4 after 300 h is shown in Figure 9. There is a 25 µm + 25 µm banded shallow pit and raised nodule structure. The chemistry is very similar to that presented for LSHR and N5. The inner layer is Al-Cr-O rich, with two S-rich bands additionally at the interfaces with the alloy and the upper layer. The upper layer consists primarily of Ni-O, with Co-O again at the outermost surface.

**Figure 10:**
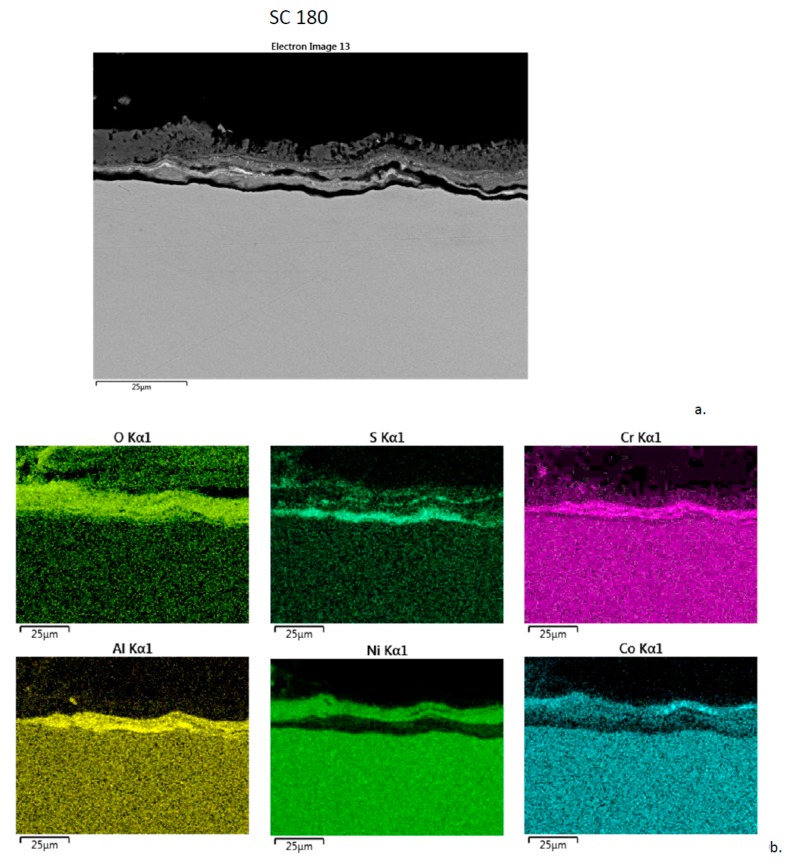
Cross-section of SC 180 sample after 300 h LTHC at 700 °C. (**a**) SE image showing two primary layers. (**b**) Elemental rasters for O, S, Cr, Al, Ni, Co showing inner Cr, Al, S bands and outer Ni, Co bands.

The corrosion product produced on (J) SC 180 after 300 h is shown in Figure 10. There is a thin 25 µm uniform dual-layer structure. The chemistry is very similar to that presented for LSHR, N5, and CMSX-4. The inner layer is Al-Cr-O rich, with S primarily at the interface with the alloy and some within the mid-layer. The upper layer consists of Ni-O, with some Co-O noted toward the outer surface.

#### 3.4.2. Experimental LDS SX Superalloys

**Figure 11:**
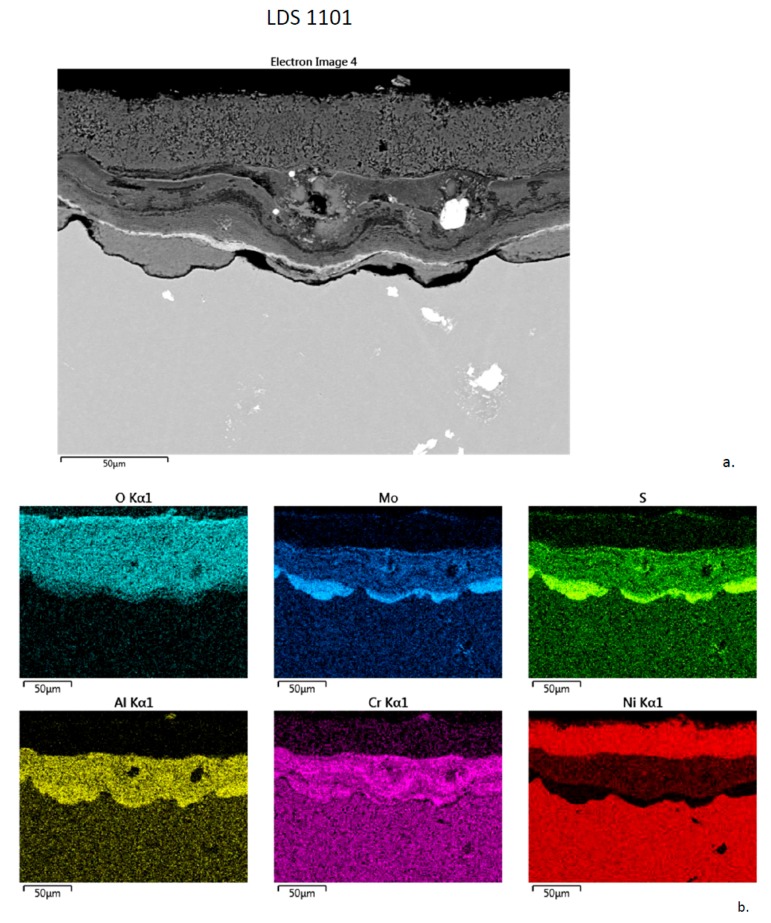
Cross-section of LDS 1101 sample after 300 h LTHC at 700 °C. (**a**) SE image showing complex corrosion layers and internal carbides. (**b**) Elemental rasters for O, Mo, S, Cr, Al, Ni showing inner Al, Cr-S cusps, intermediate Al, Cr-O band, and outer Ni band.

The (E) LDS 1101 alloy (5Cr-10Co-7Mo-3Re) shows a thick 50 + 50 µm banded dual layer with a thick distinct Ni (Co)-O scale on top of a thick Cr, Al-S, O inner layer, as shown in Figure 11. S was especially concentrated in the cusped interfacial layer. The S k_α_ (2.31 keV) and Mo L_α_ (2.29 keV) maps appeared identical, so that the apparent Mo interface concentration was likely an artifact due to S k_α_ and simply reflects the Al, Cr, S band shown for the previous alloys. The converse may be true for the uniform ‘sulfur’ (i.e., Mo) background concentration in the substrate. Bright Ta(W) borides carbides were also observed.

**Figure 12:**
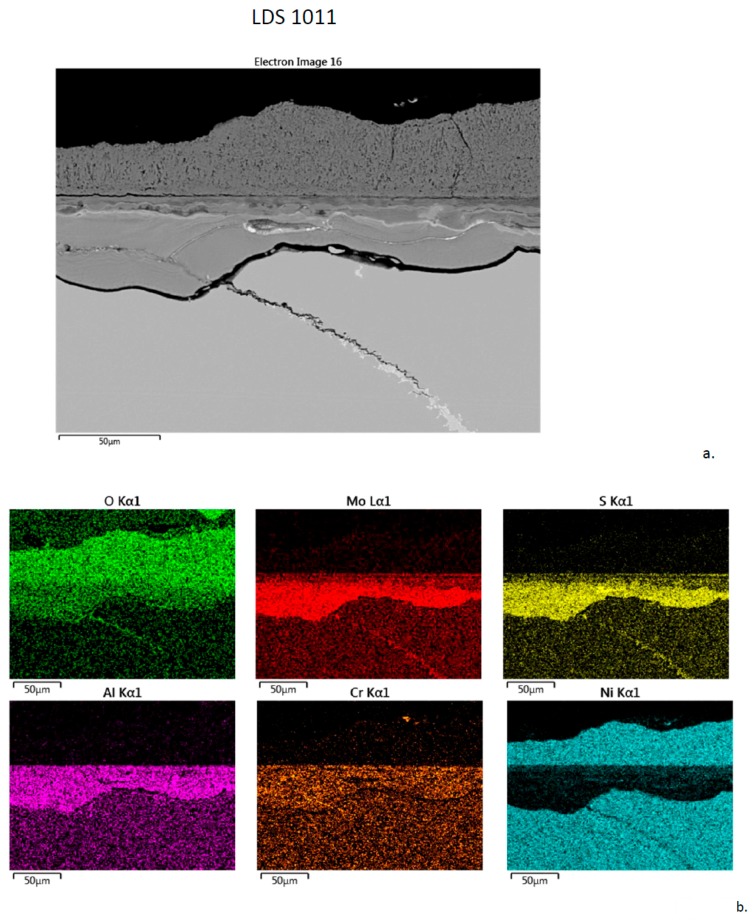
Cross-section of LDS 1011 sample after 300 h LTHC at 700 °C. (**a**) SE image showing thick distinct layers. (**b**) Elemental rasters for O, Mo, S, Al, Cr, Ni showing inner Al-Cr-S band and outer Ni-O band.

The same is true for the (F) LDS 1011 (5Cr-0Co-12Mo-3Re) alloy, as shown in Figure 12. Here the distinct boundary between the 50 + 50 µm inner/outer layers appears to be that of the original alloy surface. The Al-Cr-O-S inner layer is again highly delineated. S appears to be penetrating a long crack or bright carbide/boride boundary within the alloy.

**Figure 13:**
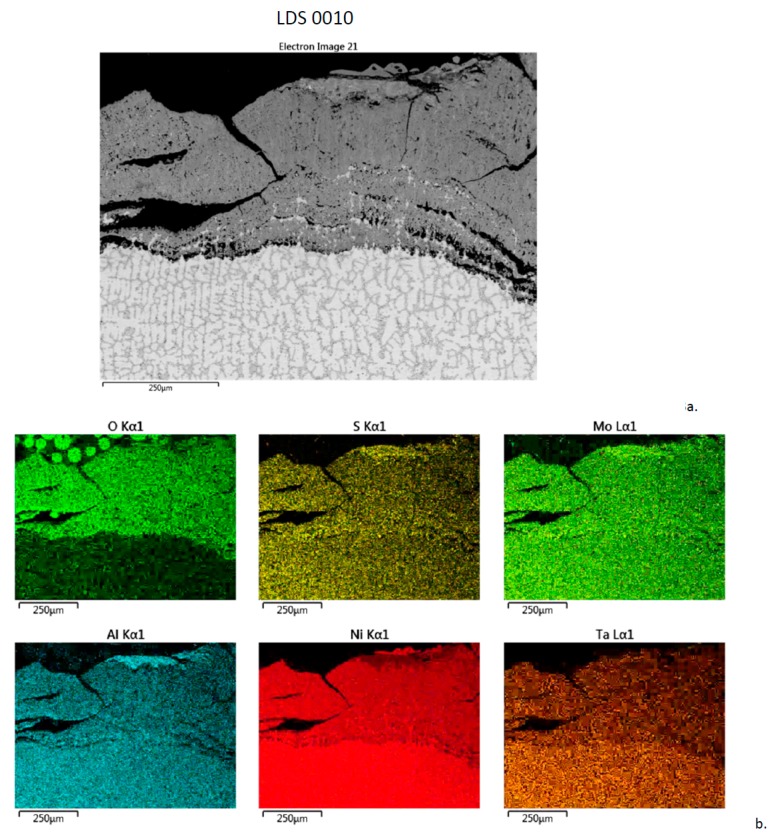
Cross-section of LDS 0010 sample after 300 h LTHC at 700 °C. (**a**) SE image showing extremely thick, cracked layer. (**b**) Elemental rasters for O, S, Mo, Al, Ni, Ta showing uniform chemical composition across the corrosion product.

Finally, an enormous, 500 µm thick, cracked corrosion product is present on the high-Mo (G), no Cr, LDS 0010 (0Cr-0Co-14Mo-0Re) alloy, Figure 13. It is primarily a thick Ni-O scale with uniform chemistry throughout. Again, S k_α_ peaks overlap with Mo L_α_, so that map, showing S within the substrate, is likely an artifact. An unusual dendritic, cored base alloy structure is also evident.

## 4. Discussion

An overall comparison of the materials can be seen in the bar chart, Figure 1. Here the final weight of the 300 h test series is compared for all 10 materials and is typical of the duplicate sample behavior in the individual plots. It is demonstrated that the Cr_2_AlC MAX phase materials as a class represent a low weight change category compared to all the Ni(Co) base superalloys. Most of the superalloys showed excessive weight loss. Only the (G) LDS 0010 highest Mo, no Cr, superalloy maintained excessive weight gain. The individual plots also showed a tight distribution of curves for the Cr_2_AlC samples, very close to that expected from the cumulative salt coatings every 50 h cycle (3 mg/cm^2^). The superalloys, however, as a group show many bifurcated trends of gains and losses, consistent with irregular scale spallation events. The cracked blue surface scales agree with the large gains/losses exhibited by most of the Ni-base superalloys. All the superalloy materials showed some salt flow, wetting, and reaction on the underside of the 300 h samples. Material loss after 500 h at 700 °C by the Cranfield metrology approach [8], showed ~225 μm for the LSHR superalloy, but indicated as low as ~25 μm for the best (purest) Cr_2_AlC material [4].

The elemental rasters further elucidated the LTHC behavior. The corrosion layers on the (D) LSHR disk superalloy, the commercial single crystal superalloys (H,I,J), and the high-Mo (LDS) superalloys (E,F,G) all exhibited a banded or two-layer structure. The upper layers were rich in Ni-O, with Co-O in the outermost regions. The inner layers contained Al-Cr-S-O. In some instances, S was further concentrated between the two layers or at the alloy interface. The exception was the highest 12% Mo, 0% Cr LDS 0010 alloy (G) that showed uniform Ni-Al-Mo concentrations throughout a massively thick Ni-O scale. The other two LDS alloys showed Al, Cr, S concentrated in the inner layer or at the alloy interface.

(Other elements Ti, Re, Ta, W, were generally at low levels in the scales indistinguishable from the bulk of all the superalloys. Occasionally, high Ta intensity was observed and associated with particulate carbides in the alloy).

It is likely that all the Ni(Co) superalloys followed typical low temperature hot corrosion mechanisms by forming K, Na, Ni, Co eutectic sulfates with melting points under 700 °C. This allowed for rapid dissolution of Ni, Co-oxides and re-precipitation at the outer surface. It is presumed that the inner Al, Cr-rich inner scale layers were discontinuous and relatively non-protective, since no major curtailment of the reaction kinetics was observed. The role of Mo in forming dissolved Na_2_MoO_4_ and accelerated Type I hot corrosion is well known.

While the present study was limited in scope to primarily screening, is it useful to compare to previous results for mechanistic insights. In the present study, the level of 300 ppm SO_2_ in the gas was aggressive enough to produce overall (uniform) surface attack. That is, pitting corrosion was not a distinctive feature here, as is often called out in Type II LTHC mechanisms. This is consistent with other studies, where high SO_2_ pressures result in aggressive uniform corrosion rather than just at the limited regions of pitting [12]. However, pitting might be possible at lower rates of corrosion by using less salt or lower SO_2_ content. Pits were more typical for the LSHR Ni-base alloy in simple 760 °C 40Mg_2_SO_4_-60Na_2_SO_4_ salt corrosion initially exposed to 1 atm air [1]. A detailed survey on LSHR pitting [12] found that 100 and 1000 ppm SO_2_ in O_2_ produced aggressive uniform attack with similar binary salts. However, 10–100 ppm SO_2_ levels in diluted 10% O_2_-90% Ar resulted in numerous fine touching pits. Even pure air or 20% O_2_-Ar produced pitting without SO_2_. Pure Ar gas environment did not produce LTHC of any form, presumably because NiO was not formed. Finally, pits were produced for LSHR by 650–700 °C exposure to a very low 2.5 ppm SO_2_ cover gas in a Na_2_SO_4_-23MgSO_4_-20CaSO_4_-7K_2_SO_4_ Bornstein salt [unpublished research by B. Gleeson].

The present study used the same 300 ppm SO_2_ pressure and apparatus as previous works [13]. That study examined a disk alloy, RR1000, similar to LSHR, at 700 °C, with 2 μg/h Na_2_SO_4_-2%NaCl deposits, for up to 500 h. These exposures produced retained scales nearly 50 μm thick and metal loss reaching ~90 μm. Distinct Ni(Co) rich outer and Cr-S-rich inner layers formed, as produced for LSHR and similar to those found in the present study for Rene′N5, CMSX-4, and SC180 commercial SXSA. However, the single crystal alloys, with nearly twice the Al content as disk alloys, generally exhibited more Al in the inner corrosion layers than the disk alloys. The LDS alloys presented additional features, with Mo associated with Cr-Al-S inner layers.

A mechanistic study of CMSX-4 used a similar Type II LTHC exposure [14]. Here 0.3 mg/cm^2^ Na_2_SO_4_ was sprayed on the samples and exposed to O_2_-1000 ppm SO_2_ at 700 °C for successive times up to 50 h. Two distinct Ni(Co) outer and Cr-Al-S-O inner layers formed within 15 min., totaling ~50 μm after 50 h, similar to the layered structures found in the present study. The sulfur was again concentrated at the interface between these layers and at the interface with the substrate. NiSO_4_ was identified after 30 min., but was consumed before 5 h.

In a related burner rig study up to 700 h, three superalloys were exposed to alkali-ingested natural gas fuel, producing deposits under 300 ppm SO_x_ and 8.7% H_2_O combustion products at 715–955 °C, flowing at 50 m/s [11]. At lower temperatures, Haynes 230 (Ni-Cr-W) showed less metal consumption (~18 μm mean loss) than IN939 (Ni-Co-Cr-Ti-Al, 23 μm) or IN738LC (Ni-Co-Cr-Ti-Al, 36 μm). Various complex chemical features were observed in the scales, but were difficult to generalize. Cr, Ni outermost oxide and sulfur enrichment in the central scale was reported for IN738 C, slightly different than the morphologies produced in the present study.

It is also pertinent that high-Mo alloys have recently been studied under low temperature corrosion conditions [15]. (700 °C, 100 h, 2.75 mg/cm^2^ Na_2_SO_4_, air or O_2_—1000 ppm SO_2_ to give 4.5 × 10^−3^ pSO_3_). The fundamental observation was that Mo could trigger alloy-based acidic corrosion from the formation of liquid Na_2_MoO_4_ that allows dissolution of the scale at the metal interface and re-precipitation of non-protective islands of the oxide at the gas surface. This resulted in Type II LTHC attack similar to Type I caused by the same Na-Mo-O species.

Furthermore, it was stated that liquid MoO_3_ (T_MP_ = 795 °C) could dissolve protective Al_2_O_3_ scales (Lutz et al., 2017) [15]. Some model Ni8Cr6Al6Mo alloys exhibited inner Ni, Cr, Al—oxide layers with outer NiO scales in air at 800 and 900 °C. High-Mo alloys IN 617, RR1000, and Ni8Cr6Al6Mo exhibited thick, 50–100 µm layers at 700 °C in 1000 ppm pSO_2_. Al, Cr, S-rich pits formed at the alloy interface, with NiO surface scales and inner layers of Na_2_SO_4_ sandwiched in between. Severe spallation occurred for IN-738. An inner concentration of Mo oxide and S layers occurred.

Attack was seen to increase between 2–8 wt. % Mo [15]. Therefore, it is reasonable to expect that similar Mo effects on acidic fluxing occurred in the present study. This would apply to the high-Mo LDS alloys having 7–12% Mo that exhibited significant attack. Consider also that LSHR contains 2.7 Mo, while N5 and SC180 contain ~2% Mo and CMSX-4 has just 0.6% Mo. Therefore, next to the LDS SXSA, LSHR would be expected to show the most adverse effect of Mo.

The studies above indicate many similar structures and relevant comparisons to the present results. Further insights are provided by the more in-depth mechanistic studies. The Type II hot corrosion mechanism of pure Ni has been recently investigated in an unambiguous, definitive, and scholarly manner [16]. Various stages have been identified and various roles have been assigned to the Na_2_SO_4_ salt coating and the SO_2_/SO_3_ cover gas. Ni-S liquid first formed (635 °C) at the oxide-metal interface and transformed to solid Ni_3_S_2_. NiO formed above this layer, dissolved in a Na_2_SO_4_-NiSO_4_, melt (660 °C), then later precipitated as a very porous, steady-state NiO scale. Gas transport through micro-channels was concluded to be rate controlling, since the estimated parabolic oxidation rate was 10^4^ that reported for a dense scale and yielded a 60 μm scale in just 20 h at 700 °C, compared to 5 μm formed in air. The micro-porosity was associated with gas evolution resulting from oxidation of the inner sulfide layer, similar to that proposed by Smialek for S-doped NiAl(Zr) [17]. It is therefore expected that aspects of all these features may apply to Type II LTHC of superalloys and indicate a possible origin of rapid non-protective scale growth.

Similarly, the 700 °C Type II LTHC of a CoCrAlY coating composition has been critically re-evaluated and explained by a new mechanism [18]. Here, at high (1000 ppm) SO_2_ levels, a duplex corrosion product with Co-O outer layer and inner layers of Al(Cr) oxides with sulfur have formed. Stability diagrams were used to show that liquid CoSO_4_-Na_2_SO_4_ salts are formed as a pre-requisite for hot corrosion. However, the widely accepted negative solubility gradient (Rapp–Goto model) was shown not to apply. Rather, *synergistic fluxing dissolution* processes were proposed for the complex system, when oxide solubility minima of various oxides are offset from the locally defined basicity. Here, basic dissolution of Al(Cr) oxides (at high Na_2_O activity) and acidic co-dissolution of Co-oxides (at high pSO_3_) occur in a cooperative manner. That is, the formation of Na_2_AlO_4_ (or Na_2_CrO_4_) from Na_2_SO_4_ and Al_2_O_3_ (or Cr_2_O_3_) can occur with the production of the acidic SO_3_ ion. That species then increases the acidic dissolution of CoO (Co_3_O_4_) to form CoSO_4_ and basic Na_2_O ions. The latter species completes the circuit by triggering more basic dissolution of Al(Cr) oxides, and so on.

In the present study, presumably the same *synergistic dissolution* mechanism can apply to Ni-Co-Cr-Al SXSA, with the caveat that more complex Ni(Co)SO_4_-Na(K)_2_SO_4_ low melting salt layers probably formed. Similar corrosion structures have been produced, but now with top layers of a primary Ni-oxide and secondary outermost Co-oxide. It also helps explain previously unexpected Al, Cr, S-rich inner layers.

Finally, the complex structures and mechanisms of SXSA LTHC are certainly dependent on alloy composition and environmental exposure conditions. The weight change behavior and end-of-test structures shown here are useful to categorize the degree of severity. However, specialized additional experiments would be needed to separate and conclude detailed mechanistic steps.

## 5. Summary

Commercial Rene′N5, CMSX-4^®^, and SC-180 single crystal superalloys were exposed to aggressive Type II molten (Na,K)-sulfate LTHC corrosion tests in air/300 ppm SO_2_ at 700 °C. They exhibited large weight losses, spallation, and thick corrosion layers during 300 h of testing. Scale cross sections revealed dual outer Ni(Co) and inner Al(Cr)S-rich corrosion layers. The outer layers may reflect an oxide fluxing mechanism, while the inner sulfide layers may reflect alloy sulfidation. Cooperative Na(Al,Cr)O_4_ and Ni(Co)SO_4_ formation may contribute to accelerated basic ↔ acidic *synsergistic dissolution* attack. Broad shallow pits or uniform attack morphologies were consistent with prior studies performed in high >100 ppm pSO_2_ environments. Experimental high 7–12% Mo (1101, 1011, 0010) LDS alloys trended toward more degradation, producing dual layer scales, again with Al(Cr)S-rich inner layers, or uniform excessively thick NiO.

## Figures and Tables

**Table 1:** Nominal composition of superalloys in low temperature hot corrosion (LTHC) test series. Low solvus high refractory (LSHR) disk alloy, commercial single crystals, and experimental Low Density Superalloy (LDS) high-Mo alloys.

Alloy	Al	Cr	Co	W	Mo	Nb	Re	Ta	Ti	Hf	Y
**LSHR**	3.4	12.9	21	4.3	2.7			1.7	3.7		
**Rene′N5**	6.2	7	8	5	2.0	1.40	3	7.0		0.2	
**CMSX4**	5.6	6	10	6	0.6		3	6.0	1	0.1	
**SC 180**	5.2	5	10	5	2.0		3	8.5	1	0.1	
**LDS-1101**	6.1	5	10		7.1		3	6.3			0.007
**LDS-1011**	6.1	5			12.0		3	6.2			0.005
**LDS-0010**	6.1				12.1			6.3			0.011
